# scACT: Accurate Cross-modality Translation via Cycle-consistent Training from Unpaired Single-cell Data

**DOI:** 10.1145/3627673.3679576

**Published:** 2024-10-21

**Authors:** Siwei Xu, Junhao Liu, Jing Zhang

**Affiliations:** University of California, Irvine, Irvine, California, USA; University of California, Irvine, Irvine, California, USA; University of California, Irvine, Irvine, California, USA

**Keywords:** Multimodal Integration, Cross-modality Translation, Single-cell RNA-seq, Single-cell ATAC-seq, Model Interpretability, Cycle-consistent Adversarial Training

## Abstract

Single-cell sequencing technologies have revolutionized genomics by enabling the simultaneous profiling of various molecular modalities within individual cells. Their integration, especially cross-modality translation, offers deep insights into cellular regulatory mechanisms. Many methods have been developed for cross-modality translation, but their reliance on scarce high-quality co-assay data limits their applicability. Addressing this, we introduce scACT, a deep generative model designed to extract cross-modality biological insights from unpaired single-cell data. scACT tackles three major challenges: aligning unpaired multi-modal data via adversarial training, facilitating cross-modality translation without prior knowledge via cycle-consistent training, and enabling interpretable regulatory interconnections explorations via in-silico perturbations. To test its performance, we applied scACT on diverse single-cell datasets and found it outperformed existing methods in all three tasks. Finally, we have developed scACT as an individual open-source software package to advance single-cell omics data processing and analysis within the research community.

## 1 Introduction

The recent advancements in single-cell sequencing technologies have significantly transformed genomics, allowing for the detailed measurement of molecular information from individual cells [15, 18]. This development has been pivotal in shifting our understanding of biological complexity and diversity at the cellular level [4, 7, 9, 26]. Moreover, a versatile array of single-cell tools have been routinely used to separately explore diverse molecular aspects, such as genetics, epigenetics, transcriptomics, and proteomics, enabling a more nuanced understanding of cellular functions in disease and paving the way to more effective therapeutic strategies [3, 19, 31]. However, a notable limitation of standard single-cell technologies is their ability to measure only one type of molecular information from each cell [14, 20]. This constraint presents significant challenges in fully deciphering the intricate interactions among genomic, transcriptomic, epigenetic, and proteomic factors in a comprehensive, multi-dimensional manner.

To tackle this challenge, various computational methods have been introduced to integrate multi-modal single-cell data sequenced separately without shared barcodes. For instance, several methods, encompassing both linear and nonlinear approaches, aim to project the data into a unified latent space to facilitate easier visualization and comparison across different modalities [5, 8, 22]. Subsequently, newer methods have emerged to address issues like distribution shift and partial feature overlap during the integration process, thereby enhancing the integration’s practical applicability and quality [32, 34]. Despite these advancements, a major limitation of these methods is their focus predominantly on the alignment of multiple modalities, rather than on actual cross-modality mapping. This limitation restricts a deeper understanding of the complex interactions between different molecular layers within individual cells.

In recent years, the development of single-cell multi-omics technologies has enabled the simultaneous acquisition of molecular data from multiple modalities within the same cell. This capability for paired measurements offers unparalleled opportunities to explore cellular heterogeneity through multi-dimensional information and characterizes the regulatory interplay among these modalities. Consequently, these technological advancements have spurred the creation of novel computational methods, such as Babel, which facilitate direct cross-modality translation, yielding fresh biological insights [32]. However, these methods rely heavily on joint sequencing technologies, which are not yet broadly accessible due to their more complex library preparation protocols and higher sequencing costs. As a result, fully leveraging the extensive existing single-cell atlases, which often contain data from separate modalities, remains challenging when it comes to unraveling the intricate interplay within individual cells.

Here, we propose scACT, a deep generative model to gain cross-modality biological insights from unpaired single-cell data for solve three key problems: (i) robust **alignment** of *unpaired* single-cell multi-modal data, (ii) accurate **cross-modality translation** without prior knowledge, and (iii) interpretable **in-silico perturbations** to dissect regulatory relationships across modalities. To test its performance, we applied it on diverse single-cell datasets and found it outperformed existing methods in all three tasks. We have developed scACT as an individual open-source software package^1^ to enable the scientific community for efficient processing and analysis of single-cell multiome data. Given the exponential growth of the amount of single-cell sequencing data, we anticipate that scACT will deepen our understanding of cross-modality interplay at the individual cell level and and establish further downstream analysis as well as in-silico experiments to address tough biological problems with ease.

## 2 Methods

### 2.1 Method overview

**Figure 1:**
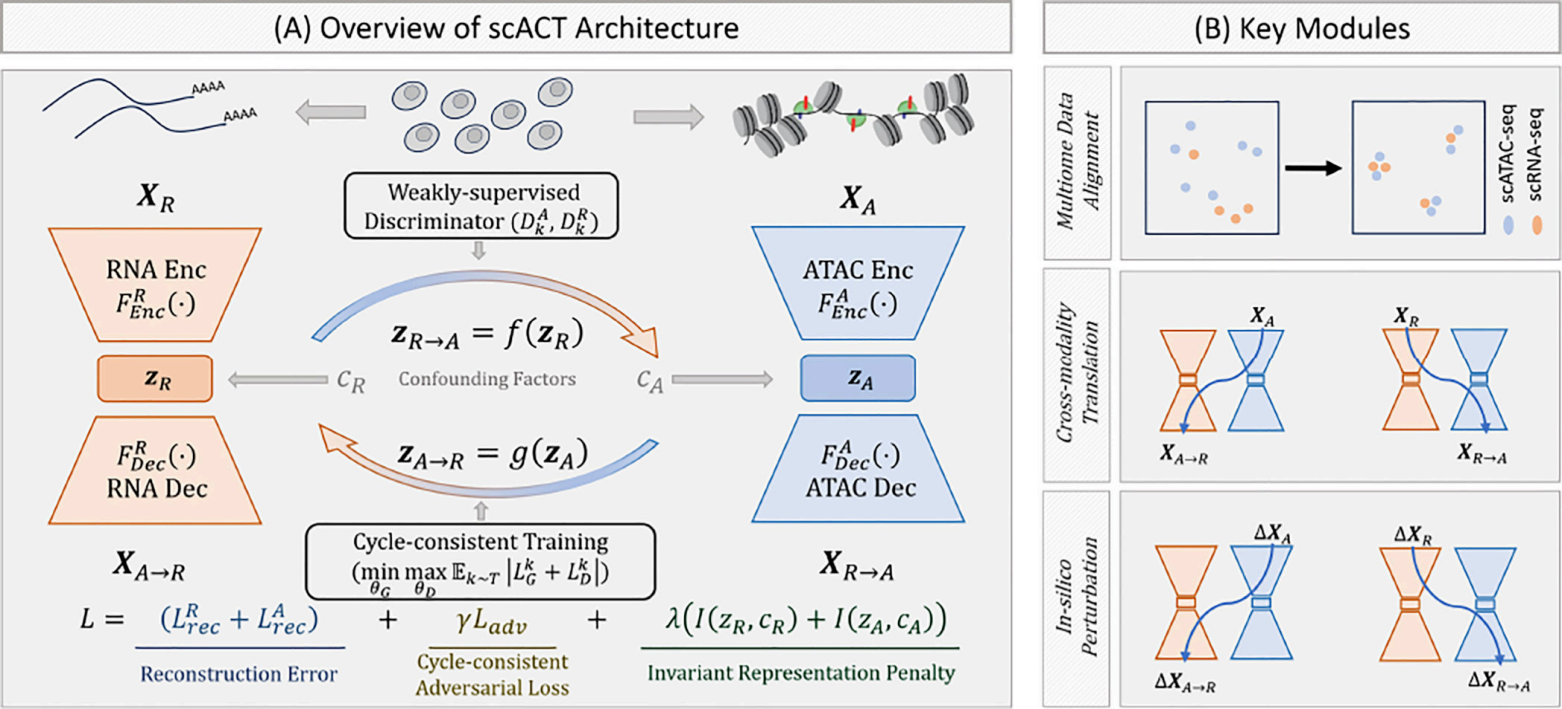
Overview, schematics, and use cases of scACT. (A) scACT’s workflow starts from the two encoder-decoder structure regularized to remove confounding factors, coupled by the two cross-modality transformation function f(⋅),g(⋅), plus the weakly-supervised discriminators $\left(D_{k}^{A},D_{k}^{R}\right.$) and the cycle-consistent training mechanism as part of the loss functions. In the illustration, scACT uses scRNA-seq and scATAC-seq from the same tissue as input; however, these two modalities do not need to have a one-to-one correspondence (no co-assay data required). (B) Key modules of scACT with multiome data alignment (top), cross-modality translation in both directions (center), and in-silico perturbation (bottom).

As shown in Fig. 1, scACT is a deep generative model with three key modules, including data alignment, cross-modality translation, and regulatory relationship inference via in-silico perturbations. Specifically, it first learns modality-specific cell embeddings to overcome the sparsity of single cell data. Then it uses cycle-consistent training to allow robust cross-modality mapping, followed by accurate cross-modality translation via the decoder structures. To further boost its performance, we added adversarial training in a weakly supervised fashion to avoid possible feature distribution shift. As a result, no paired data is needed throughout scACT’s entire learning process.

scACT is a general framework that can handle various types of single cell data, including but not limited to single-cell trancriptomics, epigenetics, proteomics. In the following sections, we used the example of scRNA-seq and scATAC-seq as examples.

### 2.2 Module 1 – multi-modal data alignment

#### Deep autoencoder for single-modality learning.

First, we applied two deep autoencoders for scRNA-seq and scATAC-seq. Given the log-normalized scRNA-seq cell-by-gene matrix ${\text{X}}_{R}\in {\text{R}}^{m\times q}$ with m cells and q genes and the scATAC-seq cell-by-peak matrix ${\text{X}}_{A}\in {\text{R}}^{n\times p}$ with n cells and p peaks, we constructed two encoders $f_{Enc}^{R}$ and $f_{Enc}^{A}$ with trainable parameters ${\text{W}}_{Enc}^{R}$ and ${\text{W}}_{Enc}^{A}$ to calculate modality-specific cell embeddings $z_{R}^{\left(i\right)}\in {\text{R}}^{k}$ and $z_{A}^{\left(j\right)}\in {\text{R}}^{k}$ of dimension k (see Suppl. Table 1 for detailed notations) as shown in Eq. 1 (see supplementary methods for details).

(1) $$z_{R}^{\left(i\right)}=F_{\text{Enc}}^{R}({\text{W}}_{\text{Enc}}^{R},x_{R}^{\left(i\right)})\;z_{A}^{\left(j\right)}=F_{\text{Enc}}^{A}\left({\text{W}}_{\text{Enc}}^{A},x_{A}^{\left(j\right)}\right)$$

Similarly, decoders $f_{Dec}^{R}$ and $f_{Dec}^{A}$ were created with trainable parameters ${\text{W}}_{\text{Dec}}^{R}$ and ${\text{W}}_{\text{Dec}}^{A}$ to reconstruct the input vectors $x_{A\to A}^{\left(i\right)}$ and $x_{R\to R}^{\left(j\right)}$ in Eq. 2.

(2) $$x_{A\to A}^{\left(i\right)}=F_{\text{Dec}}^{R}({\text{W}}_{\text{Dec}}^{R},z_{A\to A}^{\left(i\right)})\;x_{R\to R}^{\left(j\right)}=F_{\text{Dec}}^{A}\left({\text{W}}_{\text{Dec}}^{A},z_{R\to R}^{\left(j\right)}\right)$$

In our architecture, we followed biological features of ATAC-seq by first limiting hidden layer connections by chromosome with 128, 64, and 32 units in hidden layers, leading to a 20-unit dimensional reduction for the latent space. This setup, adopted in both encoder ($f_{\text{Enc}}^{R}$) and decoder ($f_{\text{Dec}}^{R}$) phases, captured the chromosomal intricacies effectively, enabling enhanced pattern recognition and accurate data reconstruction.

We adopted the mean squared error (MSE) and the binary cross-entropy loss (BCE) as the reconstruction loss functions for the scRNA-seq and scATAC-seq modalities. Combining them and using the reconstructed matrices ${\text{X}}_{R\to R}$ and ${\text{X}}_{A\to A}$, we obtain Eq. 3, the overall reconstruction loss ${𝓛}_{\text{Rec}}$.

(3) $${𝓛}_{\text{Rec}}=\text{MSE}({\text{X}}_{R},{\text{X}}_{R\to R})+\text{BCE}({\text{X}}_{A},{\text{X}}_{A\to A})$$

To address the high dimensionality and sparsity of scATAC-seq data, we followed our previous approaches [6, 33] by removing inter-chromosomal connections in ${\text{W}}_{Enc}^{A}$ and ${\text{W}}_{Dec}^{A}$.

#### Invariant representation learning to correct batch effect.

Given the confounding factors $c_{R}^{\left(i\right)}\in {\text{R}}^{a}$ and $c_{A}^{\left(j\right)}\in {\text{R}}^{b}$ (e.g., sequencing depth & batch) for each cell, we added ${𝓛}_{\text{Con}}$ to minimize the mutual information between the confounding factors and the single-modality embeddings via Eq. 4.

(4) $${𝓛}_{\text{Con}}=\sum_{i}I(z_{R}^{\left(i\right)},c_{R}^{\left(i\right)})+\sum_{j}I(z_{A}^{\left(j\right)},c_{A}^{\left(j\right)})$$

Specifically, we approximated the mutual information using its upper bound

(5) $$\begin{array}{r} I(z,c)\leq {\text{E}}_{x,c∼q(x,c)}\left[D_{KL}\left(F_{Enc}(x)\|\sum_{i^{\prime }}F_{Enc}(x)\right)\right]\\ -{\text{E}}_{z_{R}∼q_{\phi }(z|x)}\left[\text{log}F_{Dec}(z)\right] \end{array}$$

#### Encoder-mapping-decoder structure for cross-modality translation.

Following previous works [17, 36], we added two multi-layer perceptrons (MLPs) as the cross-modality mapping functions f(⋅) from RNA to ATAC and g from ATAC to RNA with trainable parameters ${\text{W}}_{RA}$ and ${\text{W}}_{AR}$ to generate the mapped embedding $z_{R\to A}^{\left(i\right)}$ and $z_{A\to R}^{\left(j\right)}$, respectively as shown in Eq. 6.

(6) $$z_{R\to A}^{\left(i\right)}=f({\text{W}}_{RA},z_{R}^{\left(i\right)})\;z_{A\to R}^{\left(j\right)}=g({\text{W}}_{AR},z_{A}^{\left(j\right)})$$

#### Weakly-supervised adversarial training to overcome feature distribution shift.

Assuming that the cell type annotations for both modalities were already known, we could used them as a weakly-supervised criteria throughout the training process of the translation. Therefore, we added an adversarial training scheme in scACT in the end. To ensure robustness in the generated modality-agnostic latent space z, we adopted a generative adversarial training mechanism [1, 11] with a discriminator using cell type annotations. More specifically, let the cell type annotation for each cell be $\text{C}=\{c_{1},c_{2},\ldots ,c_{n}\}$ where $c_{i}\in \{1,2,\ldots ,k\}$ denoting k cell types. Then, we created networks $D_{A}^{t}$ and $D_{R}^{t}$ with regard to each cell type t for the ATAC and RNA embeddings $z_{A}$ and $z_{R}$ to predict whether the embedding (encoded or cross-generated) belongs to the cell type t or not. We calculated the log-likelihood and used the evidence lower-bound (ELBO) as the adversarial training loss shown in Eq. 7,

(7) $${𝓛}_{D}^{k}={\text{E}}_{x∼{𝒞}^{k}}[\text{log}D_{A}^{k}(z_{A})]+{\text{E}}_{y∼{𝒞}^{k}}\left[\text{log}\left(1-D_{A}^{k}\left(z_{R\to A}\right)\right]\right.\;+{\text{E}}_{y∼{𝒞}^{k}}\left[\text{log}D_{R}^{k}\left(z_{R}\right)\right]+{\text{E}}_{x∼{𝒞}^{k}}\left[\text{log}\left(1-D_{R}^{k}\left(z_{A\to R}\right)\right]\right.$$

where $C^{k}$ is the set of cells within cell type k.

#### Cycle-consistent training for robust reconstruction.

In order to learn a robust mapping, we adopted a cycle-consistency loss using the output from the discriminators and the transformation functions so that they could be trained to simultaneously fool the discriminator and keep the cross-modality mapping consistent [36]. For example, for the RNA modality, we calculate the distance (MSE) between the embedding $z_{R}^{\left(i\right)}$ and its cycle-translated embedding $g(f(z_{R}^{\left(i\right)}))$. Then, we combine the negative log probability of the translated embedding $z_{R\to A}=f\left(z_{R}\right)$ being in the same cell type k. Similarly, we used BCE for the same procedure for the ATAC part. Combining both modalities, we obtained the cycle-consistency loss using Eq. 8.

(8) $${𝓛}_{\text{G}}^{k}={\text{E}}_{x∼{𝒞}^{k}}\left[-\text{log}D_{R}^{k}\left(z_{A\to R}\right)+\text{BCE}\left(f\left(g\left(z_{A}\right)\right),z_{A}\right)\right]\;+{\text{E}}_{\text{y}∼{𝒞}^{k}}\left[-\text{log}D_{A}^{k}\left(z_{R\to A}\right)+\text{MSE}\left(g\left(f\left(z_{R}\right)\right),z_{R}\right)\right]$$

#### Overall objective function.

Therefore, the overall generative adversarial training process could be summarized in the following loss function:

(9) $${𝓛}_{\text{Adv}}=\underset{\theta_{\text{G}}}{\text{min}}\;\underset{\theta_{\text{D}}}{\text{max}}\;{\text{E}}_{k∼T}[{𝓛}_{\text{G}}^{k}+{𝓛}_{\text{D}}^{k}]$$

where $\theta_{\text{G}}$ were the trainable parameters of encoders $f_{\text{Enc}}^{r},f_{\text{Enc}}^{a}$ and the cross-mapping layers $f,g,\theta_{\text{Dis}}$ collected parameters of all T pairs of discriminators $D_{A}^{k},D_{R}^{k}$. Then the overall cross-modality mapping objective is denoted in Eq. 10.

(10) $$𝓛={𝓛}_{\text{Rec}}+\gamma {𝓛}_{\text{Adv}}+\lambda {𝓛}_{\text{Con}}$$

Within the loss, γ and λ are hyperparameters to weight the adversarial and confounding loss.

### 2.3 Module 2 – cross-modality translation

In order to translate to the RNA modality from ATAC, given the input vector $x_{A}^{\left(i\right)}$, scACT generated the mapped RNA embedding $z_{A\to R}^{\left(i\right)}=g(F_{Enc}^{A}({\text{W}}_{Enc}^{A},x_{A}^{\left(i\right)}))$ (Eq. 1,6). Then, the RNA decoder generated the translation $x_{A\to R}^{\left(i\right)}=F_{Dec}^{R}({\text{W}}_{Dec}^{R},z_{A\to R}^{\left(i\right)})$ (Eq. 2). Translation from RNA to ATAC followed similar workflow and is summarized in Eq. 11 (see supplementary methods for details).

(11) $$z_{R}^{\left(i\right)}=F_{Enc}^{R}({\text{W}}_{\text{Enc}}^{\text{R}},x_{R}^{\left(i\right)})\;z_{R\to A}^{\left(i\right)}=f(z_{R}^{\left(i\right)})\;p_{R\to A}^{\left(i\right)}=F_{Dec}^{A}({\text{W}}_{\text{Dec}}^{A},z_{R\to A}^{\left(i\right)})\;x_{R\to A}^{\left(i\right)}∼\text{Bernoulli}(p_{R\to A}^{\left(i\right)})$$

Note that a bernoulli sampling step was required to generate the binarized translation to the ATAC modality.

### 2.4 Module 3 – in-silico perturbations

Based on scACT’s flexible encoder-mapping-decoder framework, we can easily conduct in-silico perturbations to obtain key regulatory insights in a cell-specific manner. Specifically, we used the concept of Integrated Gradient [29]. Given a binary input $x_{A}^{\left(i\right)}$ from the ATAC modality $x_{A}^{\left(i\right)}$ and its baseline value ${\tilde{x}}_{A}^{\left(i\right)}$ where the baseline value would be 0 for the most cases, indicating inaccessible chromatin, the integrated gradient would be:

(12) $$\text{IntegratedGradient}(x_{A}^{\left(i\right)})=({\text{x}}_{A}^{\left(i\right)}-{\tilde{\text{x}}}_{A}^{\left(i\right)})\;\times \int_{\alpha =0}^{1}\;\frac{\partial F({\tilde{\text{x}}}_{A}^{\left(i\right)}+\alpha ({\text{x}}_{A}^{\left(i\right)}-{\tilde{\text{x}}}_{A}^{\left(i\right)}))}{\partial {\text{x}}_{A}^{\left(i\right)}}\partial \alpha$$

We used this integrated gradient score as the feature importance scores with regard to a gene to find downstream analysis and discover novel regulating elements.

### 2.5 Training details

Since the two key hyperparameters in scACT are γ and λ, we adopted a grid searching approach to fine tune them by conducting a uniform search from 0.1 to 2 for both hyperparameters. Then, the best-performing model with regard to the converged overall loss was selected. After fixing the key hyperparameters, we conducted another search on the learning rate and the batch size used in the Adam optimizer we adopted in the process. We also iteratively searched from 0.00001 to 0.001 and from 16 to 256, and selected the best combination based on convergence speed and GPU memory available to us. We iteratively updated the autoencoders and the cycle-consistency units. Specifically, we prioritized on autoencoder training for feature extraction before focusing on the training of transformation functions and discriminators, and opted for a naive GAN over Wasserstein GAN based on its superior validation performance. This iterative training strategy ensures a balance between computational efficiency and model efficacy, enabling competitive outcomes without compromising on training duration or resource consumption. Detailed training steps were illustrated in the supplementary section.

### 2.6 Datasets

We included the publicly available human peripheral blood mononuclear cells (PBMC) 10k dataset and brain prefrontal cortex (PFC) dataset with parallel scRNA-seq and scATAC-seq sequencing to test our model. During the training process, we ignored the shared barcode to mimic the unpaired single-cell data, while we use the linking information as ground truth in our evaluation processes. For all modalities, we first extracted counts using CellRanger-arc (version 2.0.2) with hg38 and default parameters (see detailed pre-processing steps in supplementary methods).

#### scRNA-seq pre-processing.

We filtered out cells with insufficient reads (< 200) or possible multiplets [10] and kept the top 3,000 highly variable genes to form the scRNA-seq matrix using Pegasus (version 1.7.1) and Doubletdetection (version 4.2). We then conducted log-normalization on the entire matrix to yield the final matrix for training. We also conducted LEIDEN clustering [30] based on the PCA results (20 dimensions) and annotated their cell types using previously-studied marker genes.

#### scATAC-seq pre-processing.

Similarly, we filtered out cells with insufficient TSS enrichment (< 2.0), poor sequencing depths (< 1000), or possible multiplets using ArchR (version 1.0.1) and default parameters otherwise [12]. The peaks were called using Macs2 (version 2.2.9.1) [35] and underwent TF-IDF algorithm [25] such that only the most informative 100,000 peaks were kept. Finally, the binarized matrix was used in the training and evaluation process. To generate the cell type annotation for the discriminator, LEIDEN clustering based on the LSI results (top 20 dimension) were also used and the gene activity scores.

#### Summary of data used in this study.

Previously, we downloaded deeply sequenced scATAC-seq and scRNA-seq co-assayed data from three frozen PFC tissues (GSE216270). After strict QC, we kept 27,414 barcodes from 5 samples with seven major cell types (excitatory, inhibitory, astrocytes, endothelial, microglia, oligodendrocytes, and OPC) using the set of marker genes proposed by Lake et al. [21].

We also use the publicly available human peripheral blood mononuclear cells (PBMC) 10k dataset from the 10x Genomics website. We kept 11,582 cells from 28 cell types as suggested on the website. Cell type annotations were adopted from the Signac vignettes [28].

### 2.7 Performance Benchmarking and Evaluation metrics

We thoroughly benchmark scACT’s performance on all three tasks, including alignment, cross-modality translation, and perturbation, against state-of-the-art methods on diverse datasets.

#### Qualitative and quantitative assessment of data alignment.

We first visualize the latent space via joint-embedding UMAP [24]. Given the embeddings from RNA and ATAC modality on the same space, we created UMAP using the umap-learn package (version 0.5.5) with cosine distance, 13 neighbors, and default parameters otherwise. It was then colored by (i) the modality and (ii) the gene expression value of certain marker genes to show global homogeneity. The same UMAP generation procedure was done for Harmony and LIGER as well for comparison.

Also, to quantitatively measure the alignment, we adopted 2 metrics for global homogeneity and 2 metrics for local homogeneity. For global homogeneity, we calculated the silhouette score and the adjusted rand index (ARI) between the generated embedding and the ground truth annotation using scikit-learn (version 1.4). For local homogeneity, we measured pairwise cosine distance and modality mixing scores. We calculated a distribution of pairwise cosine distances for any two cells within the same cell type using the embeddings generated and conducted T-test. We also calculated the modality mixing score to measure how well the two modalities were mingled together in the generated joint embedding. Given the integrated latent embedding and for each cell type k, we randomly choose one cell c. Assuming that among the 1,000 closest cells, $c_{R}$ cells were from RNA and $c_{A}$ were from ATAC, then the modality mixing score $m_{k}$ could be expressed in Eq. 13.

(13) $$m_{k}=1-|\frac{c_{A}}{C_{A}+c_{R}}-0.5|$$

In the perfect case where half of the neighboring cells were from one modality, the modality mixing score would be 1.

#### ATAC-to-RNA Translation.

We first visualize the generated scRNA-seq data by overlying the expression values of previously-studied marker genes [21] on the generated UMAP. Also, we plotted a set of cell type-specific distributions for some marker genes to evaluate the difference of the predicted gene expressions across cell types. We also plotted a heatmap showing the normalized gene expression values for more marker genes across all cell types.

#### RNA-to-ATAC translation.

Since chromatin accessibility directly controls promoter-enhancer interactions, which in turns impacts motif enrichment and hence gene expression, we calculated activation scores for certain cell type-specific motifs. We here define a motif activation score,

(14) $$a_{n}=\frac{\sum_{p\in P^{\prime }}\lambda_{p}p}{\left|P^{\prime }\right|}$$

to be a weighted average of accessibility of all peaks to the upstream of 750k base pairs of the transcription start site (TSS) and plotted a heatmap showing the activation scores for motifs of marker genes. In addition, we plot a cell type-specific motif footprint using the predicted scATAC-seq information for well-studied motifs and compare it to the ground truth.

#### In-silico perturbations.

We plot the top most important peaks along with the target gene to show the capability of the model capturing important promoter-enhancer interactions of a particular marker gene (*SATB2*). To validate the found peak-to-gene interactions with regard to cell types, we plotted relavent gene expression profiles around the gene as well as the calculated peak-to-gene linkages with the most-important peaks found by scACT.

## 3 Results

### 3.1 scACT improves both local and global homogeneity when aligning different modalities

In order to evaluate scACT extensively, we adopted various metrics on its first module, multi-modal alignment on diverse datasets. As an example, we extracted a cohort of five human prefrontal cortex brain samples with 27,414 cells with both scRNA-seq and scATAC-seq and conducted model training and evaluation with the cell-level correspondence removed. Then, the trained model was evaluated from both global and local homogeneity perspectives against other baseline models, Harmony and LIGER. Through this process, a wide spectrum of metrics, such as UMAP, silhouette score, cosine distance, and modality mixing score were used. In general, we found that scACT outperformed existing methods significantly and yielded biologically-interpretable results.

**Figure 2:**
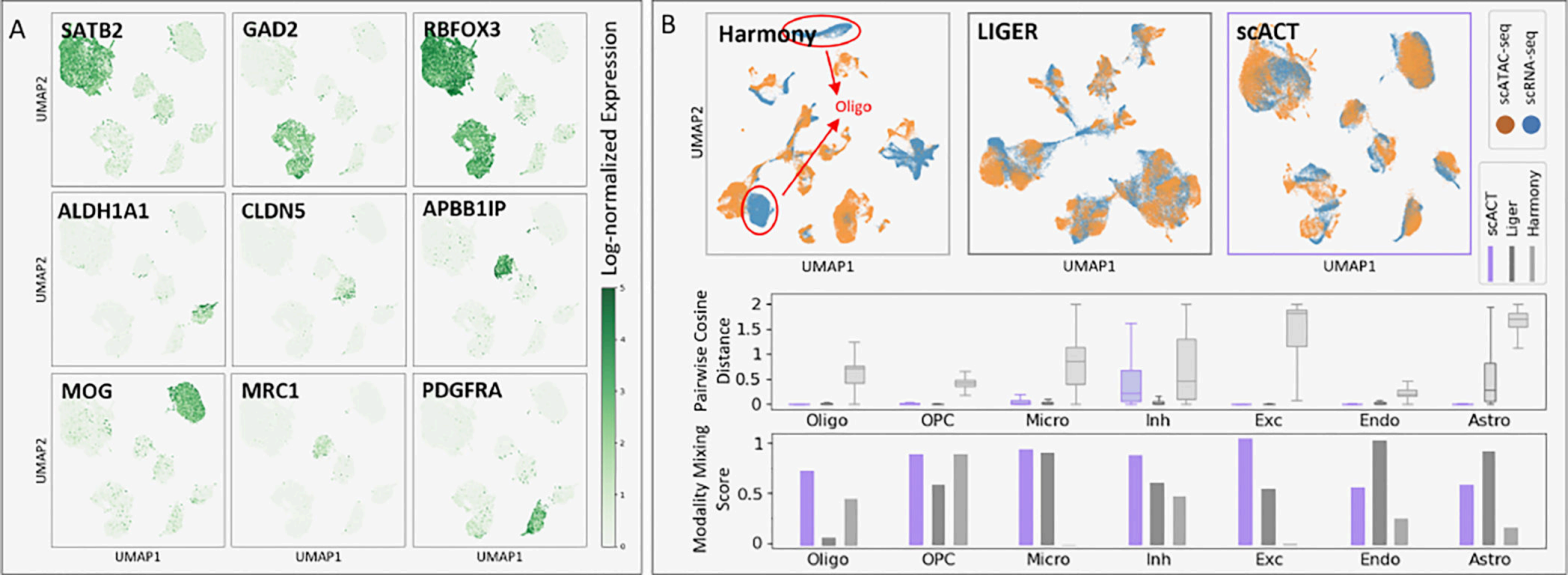
Multiome alignment performance benchmarks. (A) scACT generates better joint UMAP embeddings than Harmony or LIGER. (B) scACT’s joint embedding colored by actual gene expressions of notable marker genes show cell type consistency preserved in the embedding. (C) Pairwise cosine distance distributions within cell types show the closeness of cells within the same cell type in the generated embedding by scACT. (D) scACT mixed different modalities reasonably well.

First, for global homogeneity assessment, UMAP and silhouette scores were employed as metrics. scACT showed superior global homogeneity by generating joint UMAP representations with more meaningful and biologically-interpretable clusters (Fig. 2A). Neuronal and non-neuronal marker gene expressions overlayed on scACT’s joint embedding showed significant enrichment agreed with their cell type annotation (mean T-test p-values < 0.001) and were consistent with previous studies [21]. Also, any marker gene only illuminated one cluster, meaning that the UMAP representation was created based on cell types, not other confounding factors, such as input modalities, batch effects, or sequencing depths.

The UMAP colored by modality not only confirmed scACT’s global homogeneity, but also proved its great local homogeneity (Fig. 2B). Harmony’s embedding exhibited significant separation between modalities, lacking consistency. For instance, in the UMAP, scRNA-seq clusters, representing oligodendrocyte cells, were distant from corresponding clusters in scATAC-seq, which included endothelial and microglia cells (Suppl. Fig. 1). This discrepancy likely stems from neglecting batch effects, emphasizing experimental variations over intercellular differences. LIGER demonstrated improved cell type consistency but displayed teardrop-shaped clusters in the UMAP, suggesting susceptibility to technical factors like sequence depth. In contrast, scACT achieved better results, maintaining consistent cell type mapping and forming round or oval clusters, indicating minimal impact from confounding factors. The silhouette score further validated this, with scACT achieving a higher score (0.612) than Harmony (−0.011) and LIGER (0.510). Additional metrics (ARI and AMI) also showed clear advantages of scACT (Suppl. Table 2).

To further evaluate local homogeneity, we tested the closeness of cells within the same cell type. scACT outperformed its counterparts with a significantly lower distribution of pairwise cosine distance across all cell types (mean F-test p-value=0.0), especially in the astrocytes cells (0.026 vs. 0.426 and 0.576). Moreover, a high modality mixing score (F-test p-value< 0.05) was observed for all cell types except endothelial and astrocyte cells. In particular, in the excitatory cells, scACT was able to mix the two modalities much better than the baseline methods (0.973 vs. 0.526 and 0.081). We suspect that the less optimal performance could partially attributed to the imbalance of training data and lack of samples, but with more abundant data, we believed that scACT could deliver its local homogeneity to all cell types.

### 3.2 scACT accurately translates the ATAC modality into the RNA modality

**Figure 3:**
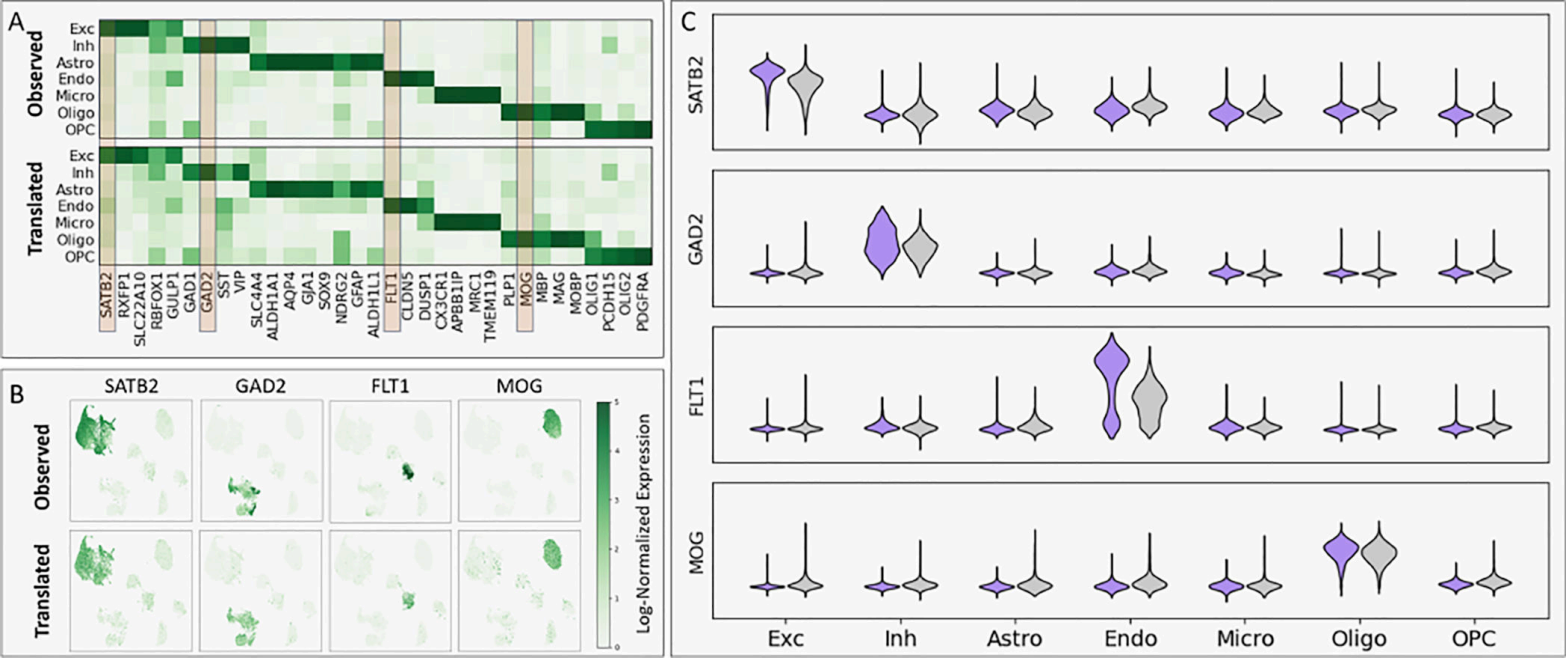
scRNA-seq prediction performance. (A) Distribution of predicted marker gene expression vs. ground truth across cell types showed great consistency and cell type specificity. (B) UMAP of actual scRNA-seq colored by actual and predicted gene expression values for the marker genes showed close resemblance. (C) normalized mean actual vs. predicted expression values captured cell type-specific variations.

Moving one-step further, we evaluated scACT’s module 2: scRNA-seq translation performance by examining marker gene enrichments and expression similarity across cell types and observed largely preserved cell type-specific signatures. First, we selected a curated list of marker genes according to previous study [21] and compared the mean expressions between cell types and between the observed and translated cohort (Fig. 3A). We found that scACT managed to preserve similar expression patterns with the observed ground truth (mean $R^{2}=0.914$), but also provided strong enrichment (mean normalized value: enriched=0.894 vs. non-enriched=0.122, mean F-test p-value< 0.001, see Suppl. Table 3 for details) with the cell type enriched consistent with previous findings [2],

Taking a closer look, we focused on four key marker genes–*SATB2* for excitatory cells, *GAD2* for inhibitory cells, *FLT1* for endothelial cells, and *MOG* for oligodendrocyte cells as they were the well-known marker genes commonly recognized. The UMAP of them agreed with our previous findings, with the translated expression highlighting the mentioned cell types, plus a high correlation between the observed and the translated ($R^{2}=0.633,0.603,0.460,0.684$, Fig. 3B). We noticed that the correlation of *FLT1* fell behind other marker genes, and suspected that the reason being the lack of training samples in that specific cell type and possible remaining multiplets (upper-left of the endothelial cluster), but overall, it yielded decent performance.

The distribution across cell types of observed versus translated profiles further verified the accuracy and correctness of scACT (Fig. 3C). A significant enrichment of these marker genes across their respective cell types was shown across all selected marker genes (mean F-test p-value< 0.0001), confirming scACT’s ability to accurately capture cell type-specific features. Plus, the distributions themselves from scACT also strictly followed the observed truth, indicating a robust learning capability. From both the distributional statistics to the actual data of each individual cell, we demonstrated its accurate translation capability from scATAC-seq to scRNA-seq.

### 3.3 scACT imputes meaningful and interpretible scATAC-seq signals

**Figure 4:**
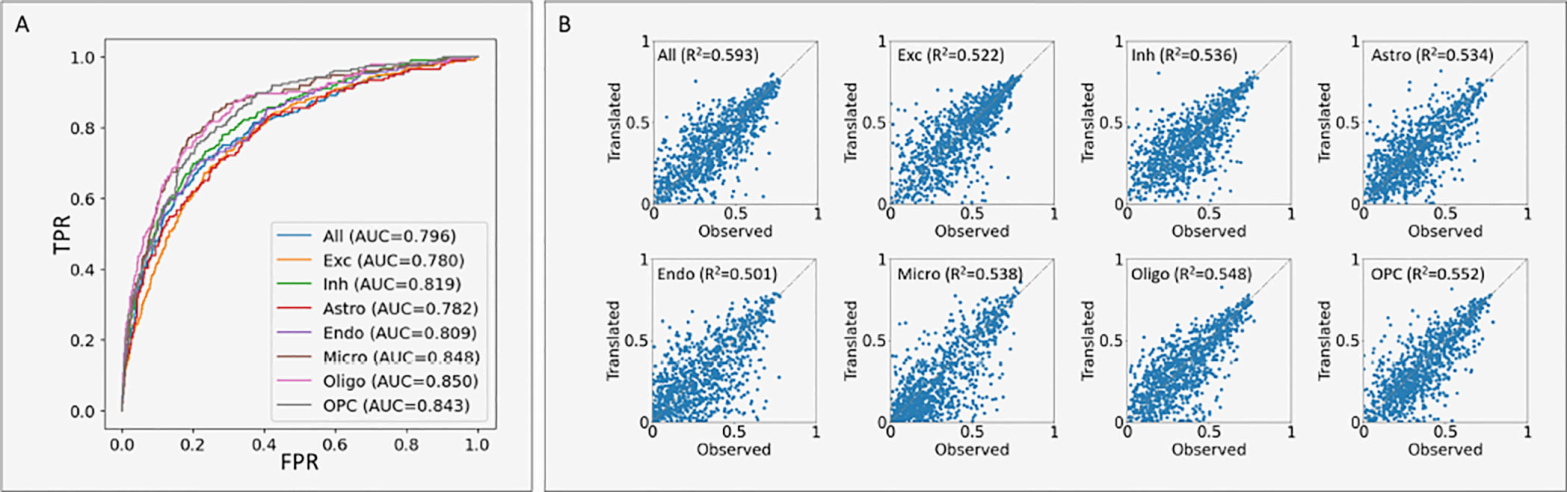
scATAC-seq prediction performance. (A) ROC curves captioned with AUCROC curves for both the entire data and each cell type. (B) Correlations between the observed and translated chromatin accessibility scores captioned with $R^{2}$ scores for both the entire data and each cell type.

Besides the robust scRNA-seq translation capability, we also assessed scACT’s scATAC-seq translation performance from the RNA modality. From a computational point of view, we can treat the translation task as a binary classification problem where for each cell, we predict the probability of each peak being accessible. In this way, we adopted the widely-used ROC curves and AUCROC scores as a key evaluation metric (Fig. 4A). Overall, scACT exhibited high accuracy, reflected in the area under the ROC curve (AUCROC=0.796). Although we found that our AUCROC was lower than other reported methods [32], we believed the reason being the different tissue of study (lymphoblastoid cells vs. brain cells) and the quality of samples used (curated cell lines vs. post-mortem tissues). We also believed that the cell-to-cell correspondence required in the other model helped largely. However, in the real world, co-assay data were at a scarce and the quality of sample varied dramatically, and thus scACT could be a better candidate providing meaningful translation. Moreover, subsetting the problem into cell type-specific clusters, we observed consistent and reasonable performance (AUCROC=0.780–0.850).

Additionally, viewing the translation problem as a regression problem in the cell type level, we extracted chromatin accessibility scores from both observed and translated data, allowing us to evaluate the correlation between them. scACT demonstrated excellent correlation ($R^{2}=0.593$) across all data and maintained consistent performance within each cell type ($R^{2}=0.501–0.548$). These results jointly highlighted scACT’s proficiency in translating scATAC-seq profiles, showcasing both accurate classification and preservation of chromatin accessibility patterns.

### 3.4 scACT enables in-silico perturbations to identify enhancer-gene linkages

**Figure 5:**
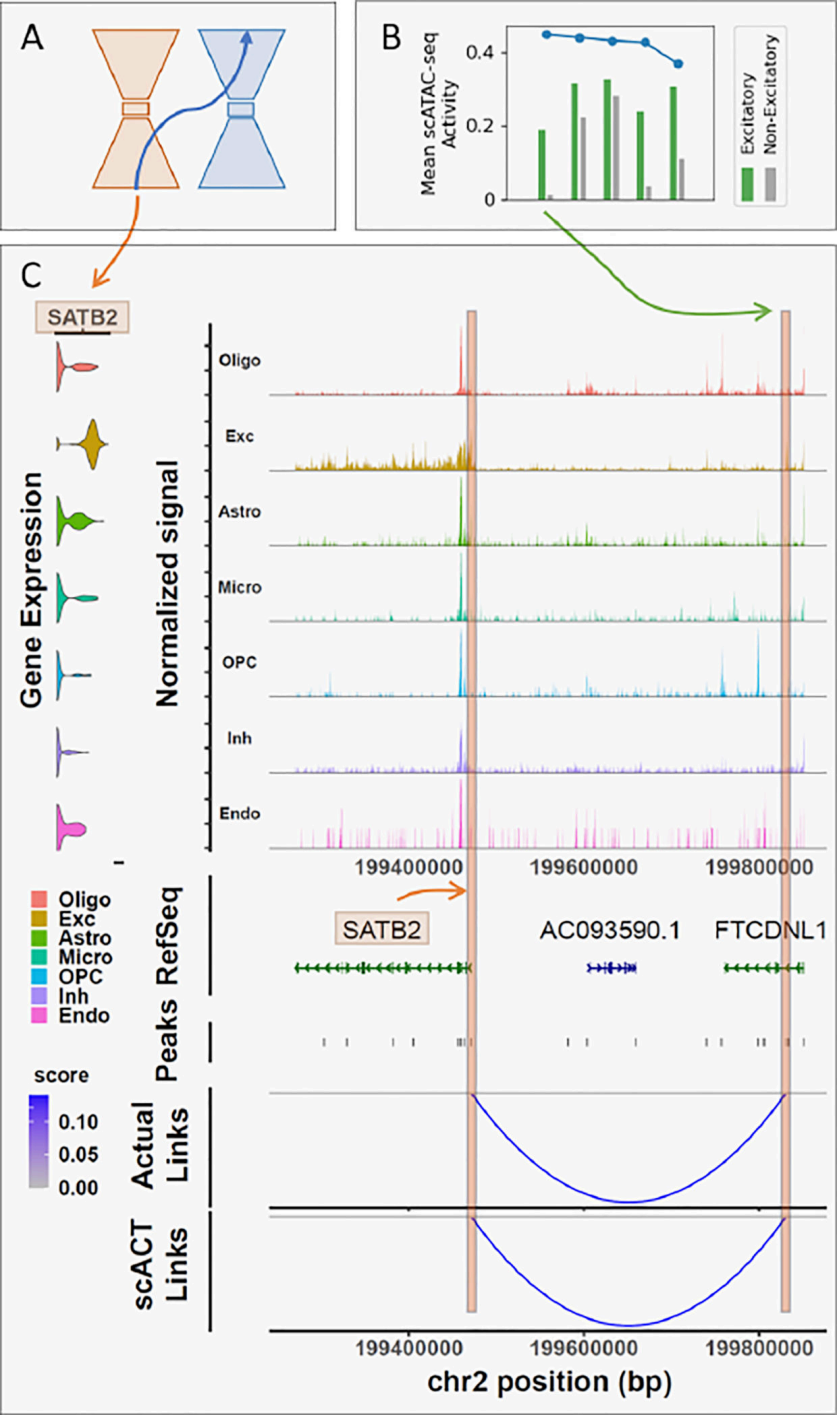
In-silico perturbation results with scACT. (A) General schematics of the module 3: perturbation framework of scACT. (B) Feature importance score (blue) plus the mean ground truth scATAC-seq activity (green and gray) for the top five peaks associated with *SATB2*. (C) Cell type-specific gene expression distribution, Tn5 insertion intensity, peak annotation, and the peak-to-gene linkages from both the ground truth and the scACT-translated scATAC-seq data.

To evaluate scACT’s in-silico perturbation capability, we focused on *SATB2*, a marker gene for excitatory cells (Fig. 5A). Feature importance scores were computed using scACT’s module 3, and the mean chromatin accessibility of excitatory and non-excitatory cells plotted alongside their respective feature importance scores for the top 5 peaks (Fig. 5B). Notably, T-tests demonstrated a statistically significant enrichment on the top peaks between these two cell groups (p-value< 0.001) and especially in the most important peak (activity=0.186 vs. 0.013, p-value=0.0), indicating the effectiveness of scACT in simulating perturbations that lead to discernible chromatin accessibility changes associated with the excitatory cell marker *SATB2*.

In addition to the test statistics across cell types, we validated the results from scACT and explored peak-to-gene linkages in both the observed and translated scATAC-seq data, focusing on the most contributing peak identified by scACTin the single-cell level (Fig. 5C). This approach allowed us to elucidate regulatory connections associated with the perturbation of *SATB2*. We found enrichment of signal for excitatory neurons on the exons and especially around the transcription start site (TSS), consistent with previous studies [16]. To our expectation, a strong enrichment was also observed for excitatory neurons on the peak identified by scACT. Further validation using the identification of peak-to-gene linkages in both observed and translated chromatin accessibility profiles (span of 100k bp; pearson test p-value< 0.05) supported scACT’s capability to not only simulate perturbations accurately, but also reveal potential regulatory mechanisms, providing valuable insights into the functional consequences with little need to conduct biological experiments.

### 3.5 scACT provides accurate alignment and translation in human PBMC

Since scACT is designed to handle various organisms and tissues, we expanded our study to evaluate its performance in the human PBMCs, which has a broader, more continuous spectrum of cell types compared to the brain prefrontal cortex data. This complexity poses additional challenges for computational models. Through extensive benchmarking, we observed that scACT maintained its robust multi-modal alignment and translation capabilities, outperforming baseline models.

**Figure 6:**
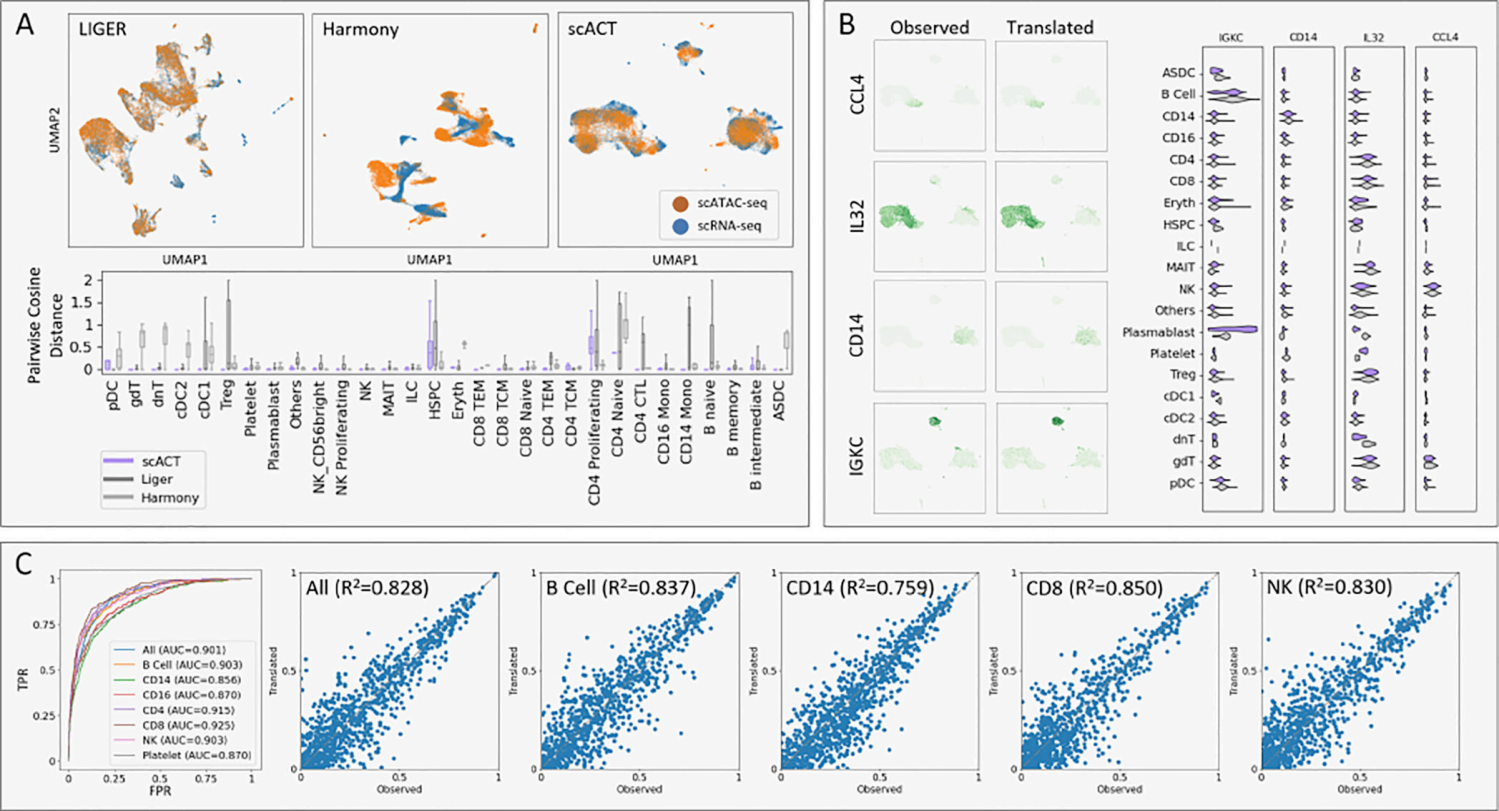
Evaluation of scACT on human PBMC multiome data. (A) Evaluation on multi-modal alignment with UMAP representations of LIGER, Harmony, and scACT (top) and comparison of pairwise cosine distances across cell types (bottom). (B) scRNA-seq translation performance with obserbed and predicted marker gene expressions (left) and the per-cell type distribution (right). (C) scATAC-seq translation benchmark with ROC curves captioned with AUCROC curves (left) and correlations between the observed and translated chromatin accessibility scores (right).

Firstly, we scrutinized scACT’s module 1, focusing on multiome alignment (Fig. 6A) and demonstrated its substantial ability to create joint embeddings. Qualitatively, scACT demonstrated global homogeneity by generating UMAP representations with more meaningful clusters. Compared to Harmony, both LIGER and scACT generated embeddings invariant to input modalities by integrating the two modalities. Moreover, scACT surpassed the baselines in cell grouping when benchmarked against annotated cell types (silhouette score: scACT=0.128, Harmony=0.125, LIGER=−0.076). Additionally, scACT’s embedding exhibited an oval-shaped structure, indicating resistance to sequencing depths and batch effects, unlike Harmony and LIGER which generated clusters with sharp edges. Quantitatively, scACT showed significantly closer clusters by cell types, especially in smaller cell types like dnT and cDC1 (mean F-test p-value< 0.001).

Next, a comprehensive inspection of scACT’s scRNA-seq translation module revealed its improved accuracy (Fig. 6B). Analysis of four marker genes (*CCL4* for NK cells, *IL32* for CD8 cells, *CD14* for CD14 cells, and *IGKC* for B cells) indicated high specificity with respect to the represented cell types (T-test p-value< 0.001), consistent with previous studies [13, 23, 27]. There was a high correlation between observed and translated matrices in most cases ($R^{2}=0.433,0.523,0.245,0.095$). For the *IGKC* gene, a potential cause for weaker performance was hypothesized to be the decreased number of cells in the B cell group and further subtypes differentiation, but in general scACT gave great performance. Zooming into cell type levels of the four marker genes revealed a clear preservation of cell type-specific signatures in expression distributions between observed and translated. Compared to out-groups, cell types corresponding to marker genes showed a significant increase in translated profiles (T-test p-value < 0.05). Overall, scACT successfully generalized its scRNA-seq translation capability to PBMC tissue.

We also benchmarked scACT’s scATAC-seq translation performance on the PBMC dataset (Fig. 6C). It effectively reconstructed chromatin accessibility from gene expressions, formulated as binary classification problems evaluated through ROC curves. Overall, scACT demonstrated high accuracy (AUCROC=0.901). When subset to larger or smaller cell type-specific clusters, it performed reasonably well (AUCROC=0.856–0.925). From a biological perspective, we sought the correlation between observed and translated chromatin accessibility per cell and per peak. scACT exhibited excellent performance with an overall $R^{2}$ value of 0.828 across all data and consistent performance in each cell type ($R^{2}=0.759–0.850$). In multiple evaluations, scACT showcased considerable translation performance from transcription profile to chromatin accessibility at the single-cell resolution.

## 4 Conclusion and Discussion

Understanding multi-modal cellular heterogeneity is crucial in genomics, and single-cell sequencing data provide great opportunities. However, deeper understanding of the hidden biology requires concurrent analysis of multiple modalities while paired co-assay data are scarce. Plus, the analysis of unpaired single-cell multi-modal data presents significant challenges. In this context, we introduced scACT, a comprehensive model designed to address these challenges by excelling in multi-modal alignment, cross-modality translation, and in-silico perturbation. Our model is particularly adept at handling unpaired single-cell multi-modal datasets, offering a powerful solution to unravel complex biological interactions.

Extensive benchmarking on diverse datasets, with a focus on brain prefrontal cortex data, demonstrated scACT’s superior multiome alignment performance compared to existing methods such as Harmony and LIGER, in preserving both global and local homogeneity in the generated joint embeddings. Plus, we showcased scACT’s proficiency in cross-modality translation in two directions: from scATAC-seq into scRNA-seq with multiple gene-level analyses and from scRNA-seq to scATAC-seq using discriminative and regressive metrics. Focusing on *SATB2* as a case study, scACT accurately conducted in-silico perturbations, as evidenced by statistically significant enrichment in chromatin accessibility changes associated with excitatory cells, aiding in understanding regulatory mechanisms as well as reducing the reliance on labor-intensive and time-consuming biological experiments Moreover, scACT demonstrated its applicability not only in brain cells, but also in other diverse tissues, such as human PBMCs, maintaining accuracy and consistency in all its modules

In summary, scACT offers a comprehensive solution for analyzing unpaired single-cell multi-modal data, providing advancements in multi-modal alignment, cross-modality translation, and in-silico perturbation using a weakly-supervised approach with cycle-consistency. The model’s robust performance across diverse datasets and tissues highlights its potential impact on advancing our understanding of complex biological systems. Currently, much more single-modality data are available with relatively low cost and high quality, whereas the emerging joint sequencing technology needs time and effort to mature. We believe that scACT can help the community in robust and accurate mapping, translation, perturbation, and further downstream analysis more effectively using more cohorts of data with higher quality to unlock more intriguing biological mechanisms and regulatory insights of different tissues and organisms.

## Supplementary Material

Supplementary Material

## CCS Concepts

• Applied computing → Computational biology; • Computing methodologies → Knowledge representation and reasoning.

## Footnotes

1
https://github.com/aicb-ZhangLabs/scACT
