# Supplementary material for: scACT: Accurate Cross-modality Translation via Cycle-consistent Training from Unpaired Single-cell Data
Source: Proc ACM Int Conf Inf Knowl Manag. Author manuscript; Available in PMC 2024 Dec 3. (PMC11611688; doi:10.1145/3627673.3679576)
Supplement: Supplementary Material [file NIHMS2035647-supplement-Supplementary_Material.zip › scACT_SupplMethods.pdf]

# A Supplementary Methods

## A.1 Model details

*A.1.1 scATAC-seq autoencoder details.* In our model architecture, we implemented a more intricate design for the autoencoder dedicated to the ATAC modality, denoted by $f^R_{\text{Enc}}$ for the encoder and $f^R_{\text{Dec}}$ for the decoder. To create an effective representation of the open chromatin peaks, we initiated the process by grouping these peaks according to their respective chromosomes, thereby ensuring that the spatial information and regulatory interactions within each chromosome are preserved. Within this framework, we established intra-chromosomal connections, employing separate neural network structures for each chromosome. At each hidden layer of these networks, we incorporated 64 and 32 units to capture the complexity and nuances of the chromatin landscape. Subsequently, we concatenated the output from all hidden layers and applied a final fully connected layer to reduce the dimensionality to 20, forming the latent representation. This elaborate design allows the model to capture intricate patterns and relationships within each chromosome, enhancing its ability to discern fine-grained details in the ATAC modality. The decoder mirrored this structure, utilizing a complementary design to faithfully reconstruct the input information from the condensed latent space.

*A.1.2 Cross-modality mapping details.* Starting from the 20-unit latent embedding obtained for each modality, we introduced an additional hidden layer with 128 units to further enrich the representation. This intermediate layer serves as a bottleneck for information integration, enabling the model to capture more complex relationships and dependencies within and between modalities. The inclusion of a hidden layer with a higher dimensionality facilitates the extraction of abstract features that contribute to a more comprehensive and expressive representation. Subsequently, we concatenated the output of this hidden layer with the 20-unit latent space of the target modality, forming a merged feature space that encapsulates both the enriched representation and the unique characteristics of the target modality. This strategic combination allows the model to fuse information from the source and target modalities, facilitating the translation process by leveraging shared patterns and capturing modality-specific nuances.

## A.2 Training details

The training process of our model involves a staged approach, where we first focus on training the autoencoders ($F^R_{Enc}, F^R_{Dec}, F^A_{Enc}, F^A_{Dec}$) before transitioning to the translation units. This sequential training strategy allows us to leverage pre-trained autoencoders to initialize the translation modules, providing a solid foundation for subsequent learning. Specifically, we start by independently training the RNA and ATAC autoencoders, ensuring that each modality's unique features are effectively captured. Once the autoencoders have learned meaningful representations, we freeze their parameters to prevent further updates and shift our attention to training the translation functions $f(\cdot)$ and $g(\cdot)$ along with the discriminators $D(\cdot)$.

In the translation phase, the frozen autoencoder parameters act as fixed feature extractors, and the focus shifts to refining the transformation functions and discriminators. The use of a naive GAN in the adversarial loss, as opposed to the Wasserstein GAN (wGAN) loss, is a deliberate choice based on superior performance observed during validation. This choice aligns with the goal of achieving robust and effective translation while minimizing computational requirements. The iterative nature of this training approach strikes a balance between computational efficiency and model performance, allowing us to achieve competitive results without sacrificing training time or hardware resources.

## A.3 Dataset processing details

In the preprocessing pipeline for all datasets, raw FASTQ files obtained from biological experiments underwent initial processing using CellRanger-arc (version 2.0.2) with the hg38 genome as the reference. Default parameters were applied to conduct the initial quality control. Subsequently, raw count matrices were generated, and further filtering was performed using the Pegasus package (version 1.7.1) for scRNA-seq data and the ArchR package (version 1.0.1) for scATAC-seq data. Cells with insufficient scRNA-seq reads ($< 200$), scATAC-seq sequencing depths ($< 1000$), or TSS enrichment ($< 2$) were excluded.

Addressing the issue of multiplets, where cells inadvertently combined during extraction, DoubletDetection (version 4.2) was employed to filter out likely multiplets in the scRNA-seq data. The resulting scRNA-seq data and scATAC-seq data were then processed to generate raw count matrices and chromatin accessibility matrices, respectively.

To prepare the datasets for training, further processing and baseline analysis were conducted on the two matrices. For scRNA-seq data, Pegasus normalization was performed using default parameters, and the top 3,000 highly variable features (genes) were selected using the pegasus.highly_variable_features function. Principal Component Analysis (PCA) and Uniform Manifold Approximation and Projection (UMAP) were applied for visualization using the pegasus.pca() and pegasus.umap() functions. Cell type annotations were curated using an annotation pipeline with pegasus.infer_cell_types, pegasus.infer_cluster_names, and pegasus.annotate functions, leveraging tissue-specific marker genes associated with each dataset.

In the case of scATAC-seq data, due to the binary nature of chromatin accessibility (open or closed), the matrix was binarized. Visualization of initial QC results was performed using ArchR's addIterativeLSI and addUMAP functions. Feature selection was conducted using the TF-IDF method to address the dataset's high sparsity. Finally, LEIDEN clustering was performed using the addClusters function, and cell type annotations were manually assigned by overlaying marker gene enrichments on scATAC-seq clusters, capturing the most likely cell types for each cluster.
