# Supplementary material for: scACT: Accurate Cross-modality Translation via Cycle-consistent Training from Unpaired Single-cell Data
Source: Proc ACM Int Conf Inf Knowl Manag. Author manuscript; Available in PMC 2024 Dec 3. (PMC11611688; doi:10.1145/3627673.3679576)
Supplement: Supplementary Material [file NIHMS2035647-supplement-Supplementary_Material.zip › scACT_SupplFig.pdf]

A Supplementary Figures

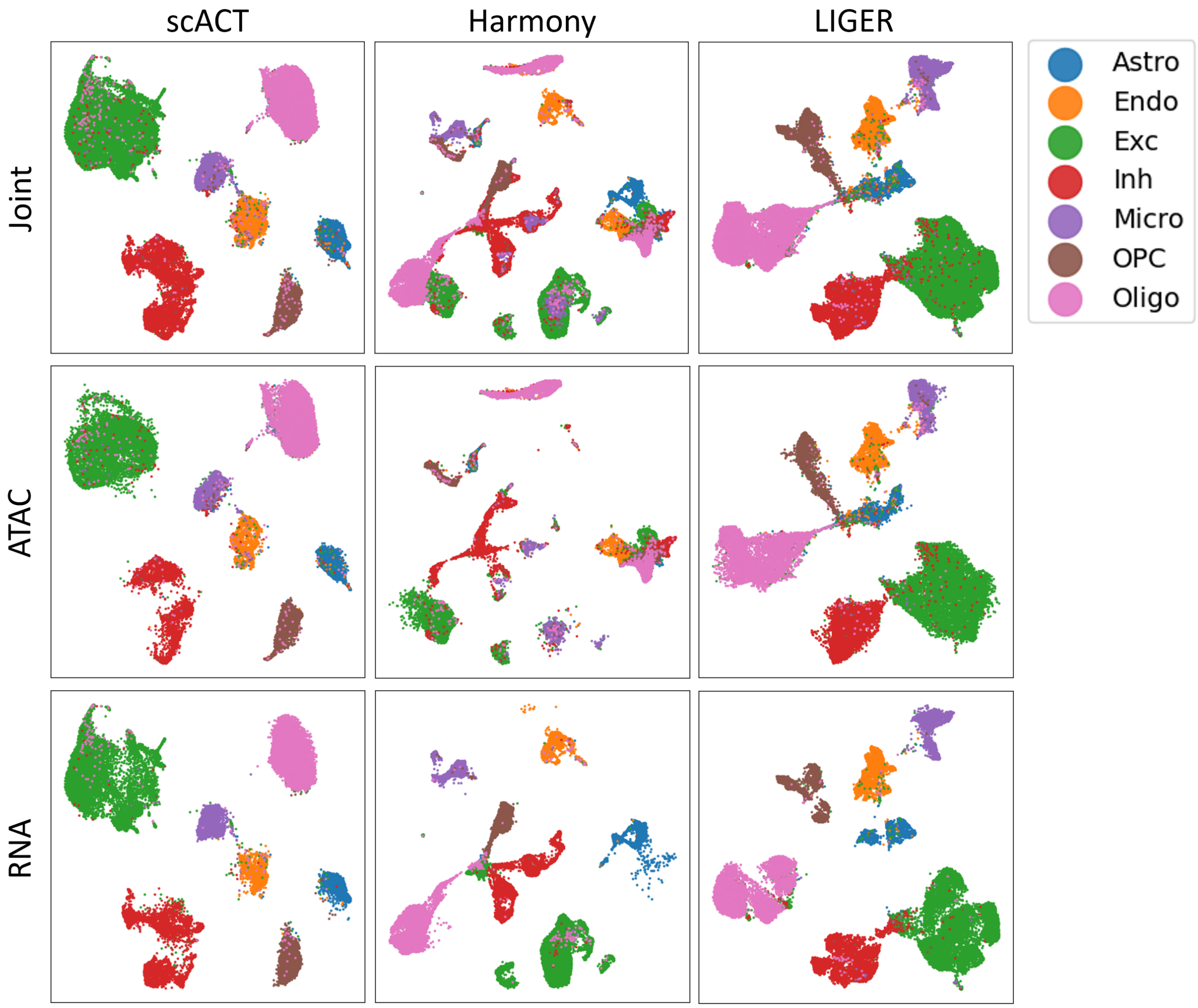

Figure 1: Joint and single-modality UMAP representations of scACT, Harmony, and LIGER colored by annotated cell types.
