# Supplementary material for: scACT: Accurate Cross-modality Translation via Cycle-consistent Training from Unpaired Single-cell Data
Source: Proc ACM Int Conf Inf Knowl Manag. Author manuscript; Available in PMC 2024 Dec 3. (PMC11611688; doi:10.1145/3627673.3679576)
Supplement: Supplementary Material [file NIHMS2035647-supplement-Supplementary_Material.zip › scACT_SupplTable.pdf]

A Supplementary Tables

Table 1: Detailed Notation Description for Methods

Name	Notation
Input Data	$x_R^{(i)}, x_A^{(i)}$
Reconstructed Data	$x_{R\to R}^{(i)}, x_{A\to A}^{(i)}$
Encoders	$F_{Enc}^{R}(\cdot), F_{Enc}^{A}(\cdot)$
Decoders	$F_{Dec}^{R}(\cdot), F_{Dec}^{A}(\cdot)$
RNA to ATAC Translation Function	$f(\cdot)$
ATAC to RNA Translation Function	$g(\cdot)$
Embeddings	$z_R^{(i)}, z_A^{(i)}$
Cross-mapped Embeddings	$z_{R\to A}^{(i)}, z_{A\to R}^{(i)}$
Cross-translated Data	$x_{R\to A}^{(i)}, x_{A\to R}^{(i)}$
Cell Type-specific Discriminators	$D_R^k(\cdot), D_A^k(\cdot)$
Confounding Factors	$c_R^{(i)}, c_A^{(i)}$
Mutual Information	$I(\cdot)$

Table 2: Detailed Alignment Performance

	Silhouette Score	ARI	AMI
scACT	**0.612**	**0.733**	**0.815**
LIGER	0.510	0.683	0.757
Harmony	-0.011	0.272	0.392

Table 3: Detailed Normalized scACT-translated scRNA-seq Expression

	Exc	Inh	Astro	Endo	Micro	Oligo	OPC
SATB2	0.865	0.098	0.177	0.329	0.198	0.229	0.104
RXFP1	0.962	0.181	0.099	0.120	0.117	0.019	0.053
SLC22A10	0.897	0.304	0.231	0.173	0.021	0.099	0.099
RBFOX1	0.584	0.552	0.239	0.252	0.213	0.171	0.398
GULP1	0.809	0.192	0.252	0.441	0.135	0.154	0.096
GAD1	0.113	0.823	0.169	0.154	0.079	0.056	0.499
GAD2	0.085	0.970	0.047	0.171	0.020	0.040	0.135
SST	0.062	0.573	0.043	0.559	0.456	0.287	0.252
VIP	0.174	0.915	0.055	0.286	0.105	0.079	0.175
SLC4A4	0.339	0.287	0.835	0.154	0.087	0.106	0.250
ALDH1A1	0.030	0.040	0.993	0.043	0.062	0.024	0.077
AQP4	0.019	0.002	0.956	0.170	0.011	0.063	0.230
GJA1	0.003	0.096	0.930	0.240	0.064	0.077	0.240
SOX9	0.122	0.157	0.940	0.259	0.003	0.071	0.066
NDRG2	0.081	0.203	0.622	0.245	0.033	0.499	0.505
GFAP	0.037	0.086	0.944	0.297	0.021	0.023	0.107
ALDH1L1	0.082	0.155	0.890	0.111	0.031	0.043	0.403
FLT1	0.088	0.092	0.168	0.962	0.120	0.046	0.116
CLDN5	0.077	0.153	0.017	0.977	0.032	0.077	0.093
DUSP1	0.167	0.103	0.360	0.792	0.424	0.055	0.149
CX3CR1	0.054	0.094	0.036	0.007	0.993	0.027	0.008
APBB1IP	0.036	0.047	0.026	0.016	0.998	0.018	0.001
MRC1	0.028	0.000	0.020	0.064	0.995	0.062	0.011
TMEM119	0.111	0.033	0.138	0.000	0.980	0.051	0.071
PLP1	0.160	0.090	0.284	0.166	0.167	0.895	0.171
MOG	0.096	0.076	0.089	0.138	0.047	0.963	0.168
MBP	0.138	0.137	0.184	0.090	0.330	0.855	0.285
MAG	0.061	0.064	0.082	0.044	0.034	0.991	0.032
MOBP	0.096	0.052	0.164	0.090	0.133	0.961	0.107
OLIG1	0.034	0.041	0.044	0.144	0.149	0.557	0.801
PCDH15	0.087	0.401	0.069	0.139	0.018	0.027	0.898
OLIG2	0.036	0.004	0.155	0.081	0.194	0.213	0.941
PDGFRA	0.025	0.119	0.077	0.029	0.023	0.027	0.988
